# GPX4‐Mediated Inhibition of Ferroptosis by Sodium Selenite Ameliorates Bone Metabolic Disorders in Ovariectomized Mice

**DOI:** 10.1111/jcmm.71414

**Published:** 2026-10-19

**Authors:** Wei Luo, Haibo Xu, Xin Liu, Yongsheng Zhang, Hao Zhou, Xiangqiang Pan, Dechun Geng, Hanliang He

**Affiliations:** ^1^ The Department of Orthopedic Surgery The Fourth Affiliated Hospital of Soochow University (Suzhou Dushu Lake Hospital) Suzhou Jiangsu China; ^2^ The Department of Orthopedic Surgery Taizhou Fourth People's Hospital Taizhou Jiangsu China; ^3^ The Department of Orthopaedic Surgery, Orthopaedic Institute, the First Affiliated Hospital, Suzhou Medical College Soochow University Suzhou Jiangsu China; ^4^ College of Chemistry Chemical Engineering and Materials Science Soochow University Suzhou People's Republic of China

**Keywords:** ferroptosis, GPX4, osteogenic metabolism, osteoporosis, selenium, sodium selenite

## Abstract

Postmenopausal osteoporosis (PMOP) and its associated fracture complications have emerged as pressing public health challenges due to rapidly accelerating global population ageing. The functional decline of bone marrow mesenchymal stem cells (BMSCs) under elevated oxidative stress is a recognised pathogenic cornerstone of PMOP, positioning redox imbalance as a critical therapeutic node. Here, we establish that sodium selenite (Na_2_SeO_3_) robustly counters oxidative insults in BMSCs, thereby restoring and enhancing their osteogenic lineage commitment. Furthermore, RNA sequencing analysis indicated that ferroptosis may be mechanistically associated with the underlying cellular responses. Ferroptosis is critically mediated by glutathione peroxidase 4 (GPX4), a selenoenzyme that functions as its principal enzymatic suppressor. Silencing GPX4 in BMSCs markedly reduced their osteogenic capacity and simultaneously upregulated ferroptotic protein expression. Notably, this impairment was not reversed by Na_2_SeO_3_ administration under conditions of GPX4 suppression. These findings suggest that Na_2_SeO_3_ enhances osteogenic differentiation primarily by upregulating GPX4 expression, thereby inhibiting ferroptosis. Thus, we have established Na_2_SeO_3_ as a potential therapeutic strategy for PMOP by identifying a previously unrecognised molecular axis through which selenium regulates bone metabolic homeostasis.

## 1 Introduction

Osteoporosis, a systemic metabolic bone disorder, is characterised by an imbalance in bone remodelling, clinically manifesting as progressive bone mass loss and bone microarchitecture deterioration [1]. These pathological alterations markedly increase bone fragility and predispose patients to fractures. Current first‐line osteoanabolic regimens predominantly utilise parathyroid hormone receptor agonists, notably teriparatide [2]. Nevertheless, their clinical application remains constrained by substantial financial burdens, mandated restrictions on treatment duration, and suboptimal patient adherence [3]. The effective prevention and treatment of osteoporosis have emerged as a pressing challenge in current clinical practice. Recent studies have highlighted the considerable potential of selenium in promoting bone regeneration, underscoring its therapeutic promise in the management of osteoporosis [4, 5]. However, the clinical implementation of conventional selenium‐based therapeutics remains challenging due to their narrow therapeutic window, resulting from dose‐limiting systemic toxicity and the paucity of robust clinical trials validating their efficacy in osteoporosis [6]. Thus, a comprehensive delineation of selenium's mechanistic involvement in osteoporotic pathogenesis is imperative to guide the development of targeted therapeutic interventions for this debilitating condition.

Ferroptosis, a distinct modality of regulated cell death mechanistically rooted in iron‐dependent, catastrophic lipid peroxidation, has emerged as a fundamental process in pathophysiology [7, 8]. In oncology, ferroptosis's capacity for potent tumour cell eradication presents a compelling therapeutic avenue, particularly for circumventing resistance mechanisms that often limit the efficacy of standard antineoplastic agents [9, 10, 11]. Moreover, ferroptosis plays a protective role in neurological disorders and ischaemia–reperfusion injury, underscoring its broad significance in the pathogenesis of diverse diseases [12, 13]. Ferroptosis is increasingly implicated as a key pathogenic determinant in the aetiology of skeletal disorders [14]. Specifically, osteoblast apoptosis induced by excessive lipid peroxidation impairs bone formation, while secondary effects promote osteoclast activation, thereby exacerbating osteoporosis progression [15]. Recent studies have demonstrated that GPX4 is the only selenoprotein capable of reducing phospholipid hydroperoxides (PLOOH) [16, 17]. Lipid‐reactive oxygen species are effectively neutralised through the GPX4/SLC7A11/glutathione axis, positioning GPX4 as a pivotal molecular hub that links selenium‐based therapeutic strategies with the regulation of skeletal homeostasis [18]. These insights highlight a novel strategy for promoting osteogenic differentiation and provide potential therapeutic targets for the prevention and treatment of osteoporosis.

Na_2_SeO_3_, an inorganic selenium compound, is one of the most widely used selenium supplements in clinical practice [19]. The physiological indispensability of the trace element selenium (Se) is met by bioavailable precursors such as sodium selenite. This inorganic compound not only corrects systemic selenium insufficiency but also exerts direct antioxidant and immunomodulatory functions [20]. Clinical evidence has indicated that sodium selenite can effectively treat and prevent selenium deficiency disorders, such as Keshan disease, and has therapeutic value as an adjuvant in cancer therapy and autoimmune thyroiditis management [21, 22, 23]. An inverse epidemiological association between selenium intake and osteoporosis incidence has been noted, with a putative mechanistic link to the element's antioxidant function in osteoblasts. Nevertheless, the molecular determinants orchestrating this protective effect remain unresolved [24]. Herein, we focused on the role of Na_2_SeO_3_ in preventing osteoblast ferroptosis and its therapeutic potential against osteoporosis. In vitro experiments revealed that Na_2_SeO_3_ mitigates oxidative stress in osteoblasts, thereby enhancing osteogenic differentiation. Furthermore, silencing of GPX4 confirmed that suppression of its expression attenuates the pro‐osteogenic effects of sodium selenite. Thus, this study was designed to interrogate the capacity of Na_2_SeO_3_ to regulate the osteogenic differentiation of BMSCs and to delineate the underlying molecular mechanisms involved, thereby providing insights into its potential therapeutic utility in bone tissue regeneration.

## 2 Method

### 2.1 Reagents and Materials

Sodium selenite was purchased from Sigma‐Aldrich (USA). BMSCs were cultured in Alpha Minimum Essential Medium (α‐MEM) and fetal bovine serum (FBS) was obtained from HyClone (USA). Phosphate‐buffered saline (PBS) was purchased from Solarbio (China). RIPA lysis buffer was purchased from NCM Biotech (China).

### 2.2 Cell Culture and Viability Assay

Primary BMSCs were procured from 6 weeks‐old Sprague–Dawley rats and expanded in α‐MEM containing 10% FBS. The cultures were maintained under standard incubation parameters in a humidified atmosphere of 5% CO_2_ at 37°C. After 5–7 days of initial adherence, cultures were monitored. When the cells reached 80% confluence, they were trypsinised and passaged. Third‐passage BMSCs were used for all subsequent experiments. Cellular viability was quantified by seeding cells at a density of 3000 cells per well into 96‐well plates. The cells were allowed to adhere overnight. Subsequently, the cultures were subjected to the designated experimental treatments for the specified durations. The in vitro intervention concentration of 0.1 μM Na_2_SeO_3_ was selected based on our preliminary cytotoxicity screening and physiological selenium tolerance profiles. At the treatment endpoint, the supernatant was aspirated, and cell viability was assessed by incubation in fresh medium containing 10% Cell Counting Kit‐8 (CCK‐8) solution for 2 h at 37°C under light‐protected conditions. The chromogenic signal was quantified by measuring absorbance at 450 nm, and the resulting optical density values were used for subsequent data analysis.

### 2.3 Alkaline Phosphatase (ALP) Assay

To evaluate early osteogenic commitment, third‐passage (P3) BMSCs were seeded into 24‐well plates at a density of 2 × 10^5^ cells per well. Following stable adhesion, the culture medium was exchanged for an osteogenic induction formulation, which was replenished every 3 days. At Day 7 of differentiation, the cultures were subjected to a triple wash with PBS (3–5 min per wash) before being fixed in 4% paraformaldehyde for 25 min at ambient temperature. After subsequent PBS rinses, enzymatic activity was visualised by incubating the cells for 1 h with a 5‐bromo‐4‐chloro‐3‐indolyl phosphate/nitro‐blue tetrazolium (BCIP/NBT) solution, protected from light. Quantitative assessment was subsequently performed by measuring absorbance at 600 nm, and data were analysed using ImageJ (NIH).

### 2.4 Alizarin Red S (ARS) Staining

Third‐passage BMSCs were seeded into 24‐well plates at a density of 1.5 × 10^5^ cells per well and cultured in osteogenic induction medium after adhesion. The medium was refreshed according to cell density, and osteogenic induction was terminated on Day 21. For quantification of mineralised matrix deposition, the culture supernatant was aspirated, and the cell layers were washed three times with PBS, with care taken to preserve nascent calcium nodules. Fixation was achieved by incubating for 15 min in 4% paraformaldehyde at ambient temperature, followed by three successive PBS rinses. Mineralised deposits were then visualised by incubating each well with 300 μL of Alizarin Red S solution for 30 min. Following removal of the staining solution and three subsequent rinses with deionised water, the cultures were immediately subjected to microscopic examination, and absorbance was quantified at 530 nm.

### 2.5 RNA Isolation and Quantitative Real‐Time PCR (qRT–PCR)

Total RNA was isolated from experimental cell populations using TRIzol reagent (Invitrogen), and its concentration and purity were subsequently confirmed using a NanoDrop spectrophotometer (Thermo Fisher Scientific). The isolated RNA was reverse‐transcribed to generate first‐strand cDNA libraries using the PrimeScript RT Master Mix (Yeasen), per the manufacturer's directions. The relative abundance of target transcripts was subsequently interrogated by quantitative real‐time PCR, executed on a CFX96 Real‐Time PCR Detection System (Bio‐Rad). Final expression values were derived using the 2^^−ΔΔCq^ method, with all data normalised to the endogenous reference gene *GAPDH*. All primer sequences utilised for this analysis are detailed in Table S1.

### 2.6 Western Blotting

For protein extraction, BMSCs were cultured in 6‐well plates and subjected to the indicated experimental conditions. Following treatment, cellular material was pelleted by centrifugation (20,000 rpm, 3 min, 4°C) and subsequently solubilised in RIPA buffer (NCM Biotech). Insoluble debris was removed from the lysates by high‐speed centrifugation (14,800 rpm, 25 min, 4°C), and the clarified supernatants were collected for subsequent analysis. Protein concentrations were determined using a BCA Protein Assay Kit (Thermo Fisher Scientific, USA), and samples were diluted with RIPA buffer to equalize protein concentrations across groups. Equivalent protein lysates were resolved via SDS–PAGE and electroblotted onto polyvinylidene fluoride (PVDF) membranes. Non‐specific sites were blocked for 1 h at room temperature (20°C–25°C) using 5% non‐fat milk in TBS. Membranes were then incubated overnight at 4°C with the following 1:1000‐diluted primary antibodies (Abcam, UK): anti‐COL1A1 (ab260043), anti‐Runx2 (ab23981), anti‐GPX4 (ab125066), anti‐SLC7A11 (ab307601), and anti‐COX2 (ab179800). The membranes were then washed and incubated with HRP‐conjugated secondary antibodies for 1–1.5 h. Protein bands were visualised by enhanced chemiluminescence (ECL) using reagents from Beyotime (China).

### 2.7 RNA Sequencing (RNA‐Seq)

Transcriptomic profiling of BMSCs subjected to H_2_O_2_ or Na_2_SeO_3_ treatment was performed using RNA‐Seq. To define the transcriptional landscape shaped by H_2_O_2_ and Na_2_SeO_3_ in BMSCs, constructed RNA libraries were subjected to high‐throughput sequencing utilising an Illumina/MGI platform. The entire sequencing pipeline, including downstream bioinformatic interrogation aimed at elucidating the underlying biological mechanisms, was performed by GENEWIZ (China) on a commercial basis.

### 2.8 Mitochondrial Membrane Potential Assay

To interrogate mitochondrial membrane potential, cultures were established in 24‐well plates (2 × 10^4^ cells/well) for subsequent designated experimental treatments. Mitochondrial polarization was assessed using the ratiometric fluorescent probe JC‐1 (Beyotime Biotechnology). Following a single wash with PBS and replenishment with fresh medium, cells were loaded with a JC‐1 working solution (0.3 mL/well) and incubated for 20 min at 37°C under light‐protected conditions. The probe solution was then aspirated, and the cell layers were washed 3 times (3 min each) with 1 × JC‐1 wash buffer freshly diluted from a 5× stock. The differential fluorescence was then visualised by epifluorescence microscopy (Zeiss, Germany).

### 2.9 Reactive Oxygen Species (ROS) Assay

The quantification of intracellular ROS was performed utilising the fluorescent probe DCFH‐DA. According to the manufacturer's protocol, a working solution was freshly prepared by diluting the stock probe to a final concentration of 10 μM. To quantify intracellular ROS, BMSCs cultured in 24‐well plates under the specified treatment conditions were loaded with the working solution of the fluorescent probe (0.3 mL/well). The incubation was conducted for 30 min at 37°C, with all manipulations performed under light‐protected conditions. Following this loading period, the cultures were washed twice with serum‐free medium (3–5 min per wash) to complete the removal of unincorporated dye. The intracellular fluorescence signals were then promptly assessed via epifluorescence microscopy (Zeiss, Germany).

### 2.10 Lipid Peroxidation Assay and GSH Assays

The extent of lipid peroxidation was indexed by quantifying its principal byproduct, malondialdehyde (MDA). Following three washes with PBS, BMSCs were lysed in RIPA buffer fortified with protease inhibitors. The lysates were subsequently centrifuged at 12,000 rpm for 15 min at 4°C, and the MDA content within the supernatants was measured spectrophotometrically at 532 nm, per the manufacturer's protocol (Beyotime Biotechnology). Concurrently, intracellular glutathione levels within the lysates were assayed using a commercial kit (Sigma, USA).

### 2.11 FerroOrange Staining

BMSCs cultured in 24‐well plates were subjected to the designated treatments to interrogate the intracellular labile iron pool. Post‐treatment, the cultures were washed three times for 3 min in Hank's Balanced Salt Solution (HBSS). The cells were subsequently loaded with a 1 μM solution of the Fe^2+^‐selective fluorescent probe FerroOrange for 30 min under standard incubation conditions (37°C, 5% CO_2_). The resultant fluorescence, directly corresponding to intracellular labile iron levels, was then visualised via epifluorescence microscopy (Zeiss, Germany).

### 2.12 Immunofluorescence Staining

BMSCs were seeded into 24‐well plates (10^5^ cells/well) and assayed according to the experimental design. For immunocytochemical analysis, the post‐treatment culture medium was aspirated, and the cells were triple‐washed (3 min each) with PBS. Cellular fixation was performed with 4% paraformaldehyde for 15 min at ambient temperature, followed by three additional PBS rinses. To minimize non‐specific antibody binding, the cell layers were then incubated in an immunostaining blocking solution (250 μL/well) for 1 h on an orbital shaker. The cultures were subsequently probed overnight at 4°C with the appropriate primary antibodies. Following three 5 min PBS washes, antigen–antibody complexes were detected by incubating the cells with fluorophore‐conjugated secondary antibodies for 1 h at 37°C in the dark. The samples were then washed three times for 10 min in PBS and mounted in an anti‐fade medium containing the nuclear counterstain DAPI. The fluorescence signals were then assessed via epifluorescence microscopy (Zeiss, Germany).

### 2.13 siRNA Transfection

Custom‐synthesized small interfering RNAs (siRNAs) were procured from GIMA Gene Co for targeted gene silencing of GPX4. BMSCs were transfected at ~50% confluence. The siRNA duplexes were first complexed with a transfection reagent in serum‐free medium according to the manufacturer's protocol, then added to the cells. The cells were subsequently incubated for 6 h at 37°C. Then, the transfection medium was aspirated and replaced with a complete, serum‐supplemented growth medium to support subsequent culture. The specific oligonucleotide sequences utilised for this knockdown are detailed in Table S2.

### 2.14 Ovariectomized (OVX) Mouse Model Establishment, Intervention, and Sample Collection

The Animal Ethics Committee of the Fourth Affiliated Hospital of Soochow University approved all animal protocols (No. 2023231033). Thirty female C57BL/6 mice (8–10 weeks old) were used. The animals were housed in a specific‐pathogen‐free (SPF) facility, maintained at 25°C and 60% humidity on a 12 h light/dark cycle, with *ad libitum* access to chow and water. Following acclimatization, the mice were randomly assigned to one of four experimental groups (*n* = 5 per group): the sham‐operated control (sham; only peri‐ovarian fat excision + saline), ovariectomy‐induced osteoporosis model (OVX; bilateral ovariectomy + saline), low‐dose Na_2_SeO_3_, and high‐dose Na_2_SeO_3_ groups. Beginning 1 week post‐surgery, mice in the low‐ and high‐dose groups received daily intraperitoneal injections of Na_2_SeO_3_ at 0.05 mg/kg and 0.1 mg/kg, respectively, for 2 consecutive weeks. These specific doses were determined based on established physiological biosafety thresholds and prior murine models evaluating trace element supplementation in bone metabolism. The control group received equivalent volumes of saline under the same schedule. Following the completion of the injection regimen, the animals were maintained under standard laboratory conditions until they were euthanized at the 8 weeks post‐surgical endpoint. Bilateral femurs, as well as the heart, lungs, and kidneys, were procured and fixed in formalin for subsequent analysis.

### 2.15 Micro‐CT Scanning and Reconstruction

High‐resolution micro‐computed tomography (micro‐CT) was used to assess the three‐dimensional morphology of the fixed bilateral femurs. The acquisition was performed using the following parameters: voxel size, 9 μm; rotation angle, 0.7°; voltage, 60 kV; current, 170 μA; and scan region, distal femoral metaphysis. Two‐dimensional images were reconstructed into three‐dimensional datasets using the system software (SkyScan RECON), followed by quantitative analysis of bone parameters. Surface reconstruction and visualisation were performed using Mimics Medical 21.0.

### 2.16 Calcein Double Fluorescence Labeling

To evaluate bone formation, mice received intraperitoneal injections of calcein (10 mg/kg) on days 10 and 2 prior to euthanasia, generating double‐fluorescent labelling of bone surfaces. Femurs were harvested post mortem and fixed in formalin. Undecalcified hard tissue sections were prepared and examined under an upright fluorescence microscope to observe the double labels.

### 2.17 Tissue Decalcification and Paraffin Embedding

Fresh tissues were fixed in formalin for 48 h, and soft and hard tissues were separated. Hard tissues were decalcified in EDTA‐NaOH solution (200 g EDTA + 22 g NaOH per 2 L PBS) using a graded schedule (early stage: every 3–4 days; late stage: every 1–2 days) until complete decalcification, followed by PBS washes. All tissues were dehydrated in graded ethanol solutions (70%, 80%, 90%, each for 2 h; 95%, 100%, each for 1 h), transitioned into n‐butanol for 8–12 h, and infiltrated twice in paraffin at 65°C for 2 h each. Tissues were embedded and rapidly frozen at −20°C. Paraffin sections (5 μm) were prepared, flattened, mounted on slides, and baked at 60°C for 1 h.

### 2.18 Haematoxylin and Eosin (H&E) Staining

For histological analysis, paraffin sections underwent deparaffinization in xylene, followed by rehydration through a graded ethanol series (70%–100%). The sections were then rinsed in deionised water. Subsequently, the nuclei were stained with haematoxylin, and the sections were differentiated in acid alcohol and blued in ammonia water. After a 5 min running water wash, the sections were counterstained with eosin. The stained sections were then dehydrated through an ascending ethanol series, cleared in xylene, and mounted using a neutral resin for microscopic examination.

### 2.19 In Vivo Biosafety Assessment and Serum Biochemistry

To evaluate the systemic biosafety of the treatments, whole blood samples were collected from the animals at the designated experimental endpoints. The collected blood was allowed to clot at room temperature, then centrifuged at 3000 rpm for 15 min at 4°C to isolate the serum. The obtained serum samples were immediately aliquoted and stored at −80°C until further analysis. Prior to biochemical evaluation, the frozen serum was completely thawed on ice. To assess hepatic and renal functions, the serum concentrations of alanine aminotransferase (ALT), aspartate aminotransferase (AST), blood urea nitrogen (BUN), and creatinine (Cr) were quantitatively measured using an automated biochemical analyser (or respective commercial colourimetric assay kits) in strict accordance with standard operating procedures.

## 3 Statistical Analysis

Data are presented as mean ± SD. Statistical analyses were performed using GraphPad Prism 9.0. Comparisons between two groups were analysed by an unpaired two‐tailed Student's *t‐*test, while multiple comparisons were evaluated using one‐way ANOVA followed by Tukey's post hoc test. Biological replicates were *n* = 3 (in vitro) and *n* = 5 (in vivo). Significance was defined as *p < 0.05, **p < 0.01, ***p < 0.001, and ****p < 0.0001 (ns, not significant).

## 4 Results

### 4.1 Na2SeO3 Enhanced the Osteogenic Differentiation of BMSCs and Alleviated Oxidative Stress in Vitro

In the CCK‐8 assay, sodium selenite had no effect on BMSC proliferation at 0.1 μM (Figure 1A). In subsequent experiments, BMSCs undergoing osteogenic differentiation were treated with sodium selenite for 3 days in the presence of 200 μM H_2_O_2_ (Figure 1B). Western blotting demonstrated that sodium selenite treatment attenuated the H_2_O_2_‐induced suppression of osteogenic markers, including RUNX2, SP7, and COL1A1 (Figure 1C,D). Subsequently, an ALP and ARS staining‐based approach was performed to further validate the effect of sodium selenite on osteogenesis. ALP staining revealed that sodium selenite markedly enhanced alkaline phosphatase activity, indicating promotion of early osteogenic differentiation (Figure 1E,F). Furthermore, ARS staining demonstrated a significant increase in mineralised nodule formation in the sodium selenite‐treated group (Figure 1G,H). Moreover, the expression levels of RUNX2 and OCN were further verified using immunofluorescence staining. Compared with H_2_O_2_, Na_2_SeO_3_ intervention significantly enhanced the fluorescence intensity of RUNX2 and OCN (Figure 1I–K), indicating that Na_2_SeO_3_ intervention markedly restored the osteogenic capacity of BMSCs impaired by H_2_O_2_ exposure. The magnitude of this recovery was demonstrably dose‐dependent, with a markedly superior therapeutic outcome observed in the high‐dose cohort.

**FIGURE 1:**
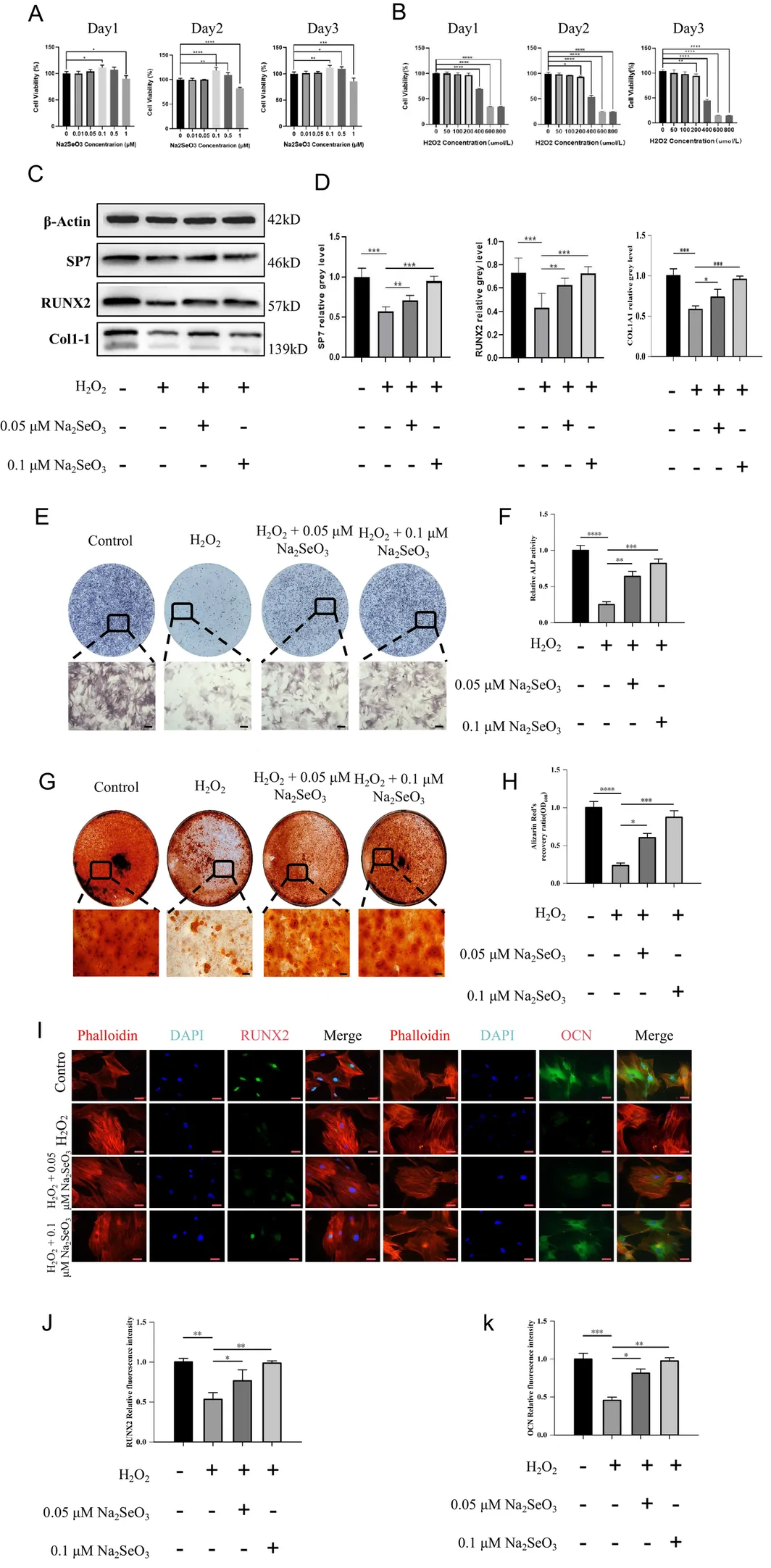
Na_2_SeO_3_ promotes extracellular osteogenesis. (A–B) Cell viability of BMSCs cultured for 3 consecutive days with different concentrations of Na_2_SeO_3_ and H_2_O_2_. (C) Protein expression levels of osteogenic markers Sp7, RUNX2, and Col‐1. (D) Evaluation of osteogenic markers depicted in panel (C). (E–G) Representative images visualizing ALP activity and ARS staining (scale bar = 100 μm). (F–H) Quantitative analysis of relative ALP activity and ARS mineralization. Data are expressed as the mean ± SD, *n* = 3; *p < 0.05, **p < 0.01 versus control; ns indicates not significant. (I) Immunofluorescence staining of RUNX2 and OCN under different treatments (blue: DAPI, red: Cytoskeleton; *n* = 3; scale bar = 50 μm). (J–K) Quantification of RUNX2 and OCN fluorescence intensity.

### 4.2 Na2SeO3 Intervention Effectively Suppressed H_2_O_2_ ‐Induced Ferroptosis in Osteogenic Cells

To identify the transcriptional basis of Na_2_SeO_3_s protective effect against H_2_O_2_‐induced cytotoxicity in osteogenic cells, we conducted comprehensive RNA‐seq analysis. Following a 72 h treatment with 200 μM H_2_O_2_ and 0.1 μM Na_2_SeO_3_, transcriptomic profiles were generated using an Illumina HiSeq 2500 platform. This unbiased approach identified a distinct transcriptional signature, comprising 329 upregulated and 336 downregulated genes (Figure 2B). Crucially, multiple independent bioinformatic analyses (KEGG, GO) converged on the ferroptosis signalling pathway, with pronounced enrichment for processes related to iron ion transport and homeostasis (Figure 2C,D). This mechanistic link was unequivocally substantiated by Gene Set Enrichment Analysis (GSEA), which demonstrated a significant suppression of the ferroptosis pathway signature in the Na_2_SeO_3_‐treated cohort (Figure 2E).

**FIGURE 2:**
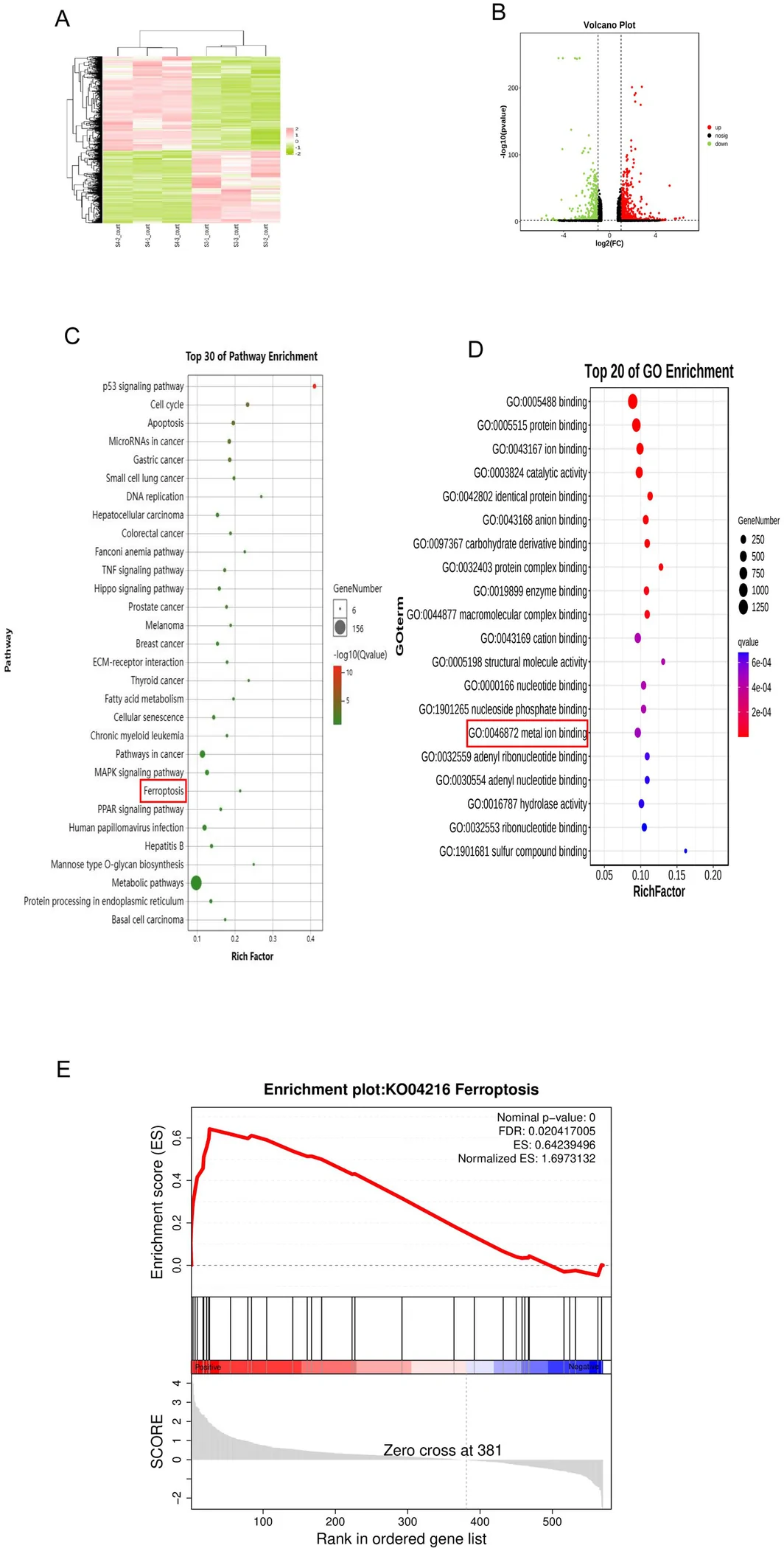
RNA sequencing of BMSCs following H_2_O_2_ and Na_2_SeO_3_ treatment. (A) Heatmap displaying differentially expressed genes across treatment groups (red: upregulated; green: Downregulated). (B) Volcano plot illustrating differentially expressed genes. (C) KEGG pathway enrichment analysis of differentially expressed genes, indicating an association with the ferroptosis pathway. (D) GO enrichment analysis showing enrichment of genes related to metal ion binding. (E) GSEA enrichment plot of the ferroptosis pathway based on RNA‐seq data.

As the transcriptomic analysis indicated ferroptosis involvement, we further elucidated the specific protective mechanism of Na_2_SeO_3_. To confirm whether Na_2_SeO_3_ mitigates H_2_O_2_‐induced damage specifically via the ferroptosis pathway, we used Ferrostatin‐1 (Fer‐1, 1 μM) as a positive control, a classical and highly specific ferroptosis inhibitor. DCFH–DA staining and corresponding quantification (Figure 3A,B) revealed that H_2_O_2_ treatment markedly increased intracellular ROS production in BMSCs. However, the administration of Na_2_SeO_3_ significantly reduced ROS accumulation in a dose‐dependent manner, exhibiting a robust antioxidative effect comparable to that of the Fer‐1 group. Consistent with the ROS alterations, both Na_2_SeO_3_ and Fer‐1 treatments markedly suppressed H_2_O_2_‐induced lipid peroxidation, as evidenced by the substantial reduction in MDA levels (Figure 3C). Furthermore, Na_2_SeO_3_ effectively restored the intracellular levels of glutathione (GSH) (Figure 3D), mitigating the depletion of the core antioxidant defence system caused by H_2_O_2_. Finally, we performed FerroOrange staining to directly assess the hallmark of ferroptosis. The results demonstrated that the intracellular labile iron overload induced by H_2_O_2_ was substantially abolished in both the Na_2_SeO_3_ and Fer‐1 groups, suggesting the successful restoration of iron homeostasis (Figure 3E). Collectively, these findings provide compelling evidence that Na_2_SeO_3_ significantly enhances the osteogenic potential of BMSCs and prevents cellular damage, primarily by acting as an effective ferroptosis inhibitor that closely mimics the functions of Fer‐1.

**FIGURE 3:**
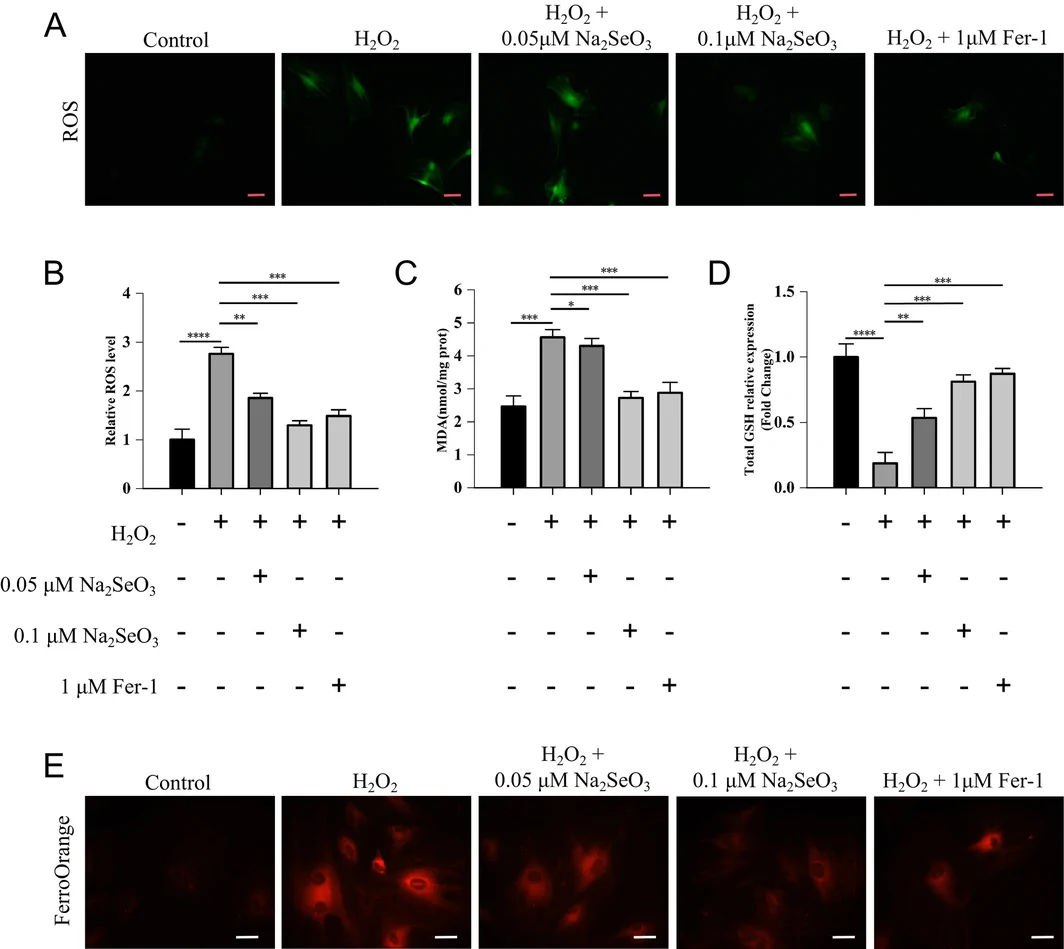
Na_2_SeO_3_ protects BMSCs against H_2_O_2_‐induced oxidative damage by inhibiting ferroptosis. (A–B) Intracellular ROS levels in BMSCs under different treatments and corresponding quantification; scale bar = 50 μm. (C) Malondialdehyde (MDA) levels. (D) GSH expression levels. (E) Representative images of FerroOrange staining (scale bar = 100 μm). Data are expressed as the mean ± SD, *n* = 3; *p < 0.05, **p < 0.01 versus control; ns indicates not significant.

Moreover, quantitative analyses of JC‐1 staining and total iron content showed a strong concordance (Figure 4A,B). Western blot and qRT–PCR analyses revealed that increasing concentrations of Na_2_SeO_3_ led to significant changes in the protein and mRNA expression levels of key regulators of ferroptosis (Figure 4C–E). In summary, our results demonstrate that Na_2_SeO_3_ functionally mitigates oxidative stress‐induced cellular damage and dysfunction and enhances osteogenic function by targeting ferroptosis in osteoblasts.

**FIGURE 4:**
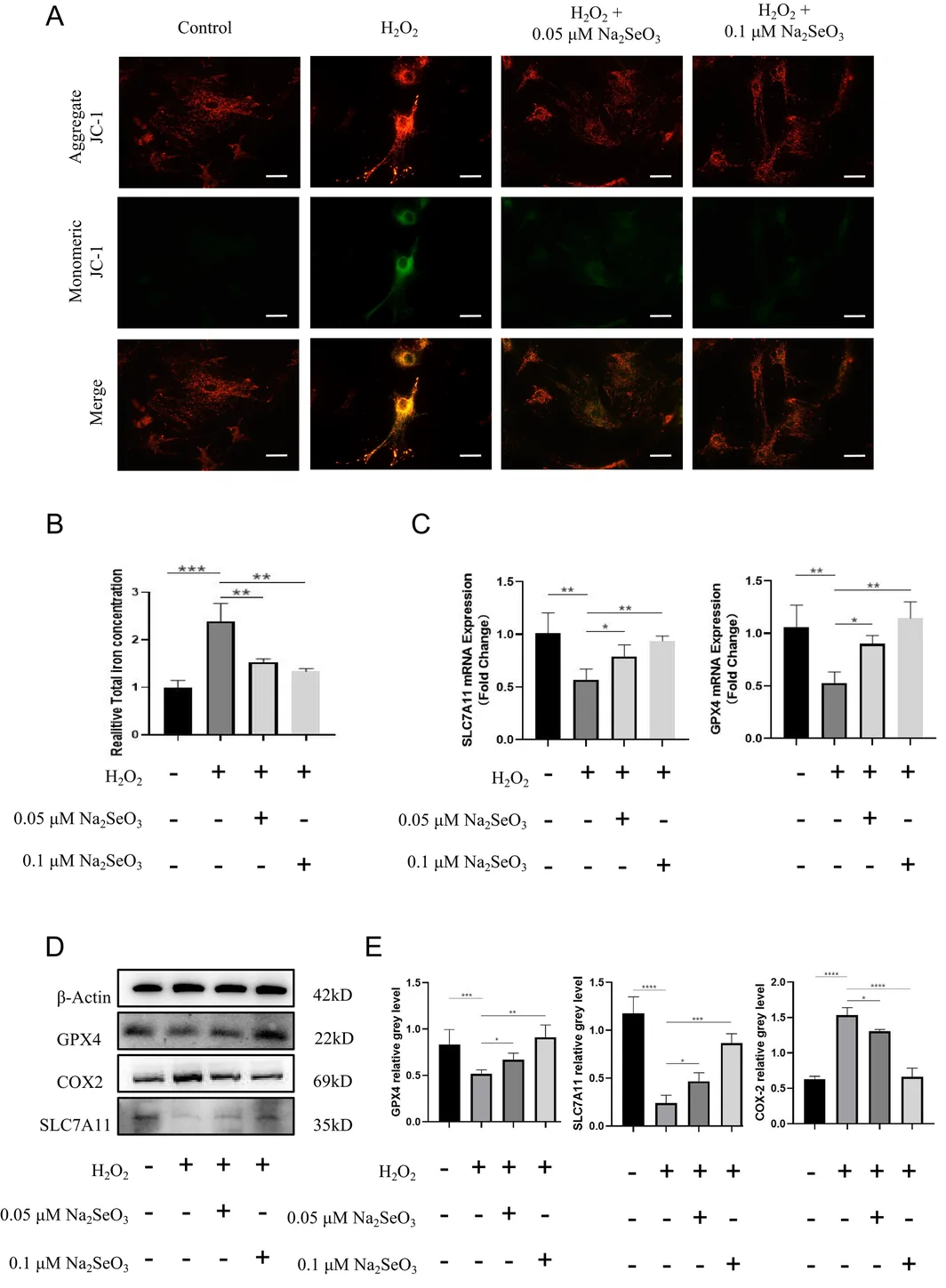
Anti‐ferroptotic effects of Na_2_SeO_3_ in vitro. (A) Representative images of JC‐1 staining (scale bar = 100 μm). (B) Total intracellular iron content. (C) mRNA expression levels of ferroptosis‐related genes. (D) Protein levels of GPX4, COX2, and SLC7A11. (E) Quantitative analysis of ferroptosis‐related proteins shown in (D). Data are expressed as the mean ± SD, *n* = 3; *p < 0.05, **p < 0.01 versus control; ns indicates not significant.

### 4.3 GPX4 Mediates the Regulation of Osteoblast Function by Na_2_SeO_3_ in Vitro

Previous studies have suggested that GPX4 plays a central role in regulating ferroptosis, with its activity determining the cellular capacity to eliminate lipid peroxides and thereby prevent ferroptotic cell death [25]. Additionally, we studied the interaction between GPX4 and Na_2_SeO_3_ using molecular docking. The optimal conformations and spatial arrangements of Na_2_SeO_3_ indicated a favourable complementarity with the GPX4 binding pocket, suggesting a potential direct interaction (Figure 5A). To experimentally validate the direct physical interaction predicted by molecular docking, we conducted a Cellular Thermal Shift Assay (CETSA). As illustrated in Figure 4B, incubation with Na_2_SeO_3_ markedly increased the thermal stability of GPX4, shifting its melting temperature (Tm) from 43.44°C to 47.25°C (ΔTm = 3.81°C). This robust thermal shift confirmed the direct physical binding between Na_2_SeO_3_ and the GPX4 protein (Figure 5B,C). Thus, we employed siRNA to silence GPX4. Subsequent western blot analyses showed that GPX4 protein expression was inhibited in both the siGPX4 and siGPX4 + Na_2_SeO_3_ groups compared with the siCtrl group, with a concurrent decrease in SLC7A11 expression. In contrast to siCtrl, Na_2_SeO_3_ treatment upregulated both GPX4 and SLC7A11 expression. Comparative analysis of osteogenic markers revealed that protein levels of RUNX2, SP7, and COL1A1 were markedly reduced in the siGPX4 group, whereas subsequent Na_2_SeO_3_ treatment failed to elicit a significant recovery of these proteins (Figure 6A–G). Nevertheless, Na_2_SeO_3_ substantially increased the expression of these markers compared with the levels observed in the untreated controls, underscoring its therapeutic potential in promoting osteogenesis. ALP and ARS staining were then performed to validate the role of Na_2_SeO_3_ in osteogenesis via GPX4 (Figure 7A–D). As expected, Na_2_SeO_3_ effectively restored the H_2_O_2_‐induced impairment of osteogenic function; however, this effect was abolished upon GPX4 knockdown.

**FIGURE 5:**
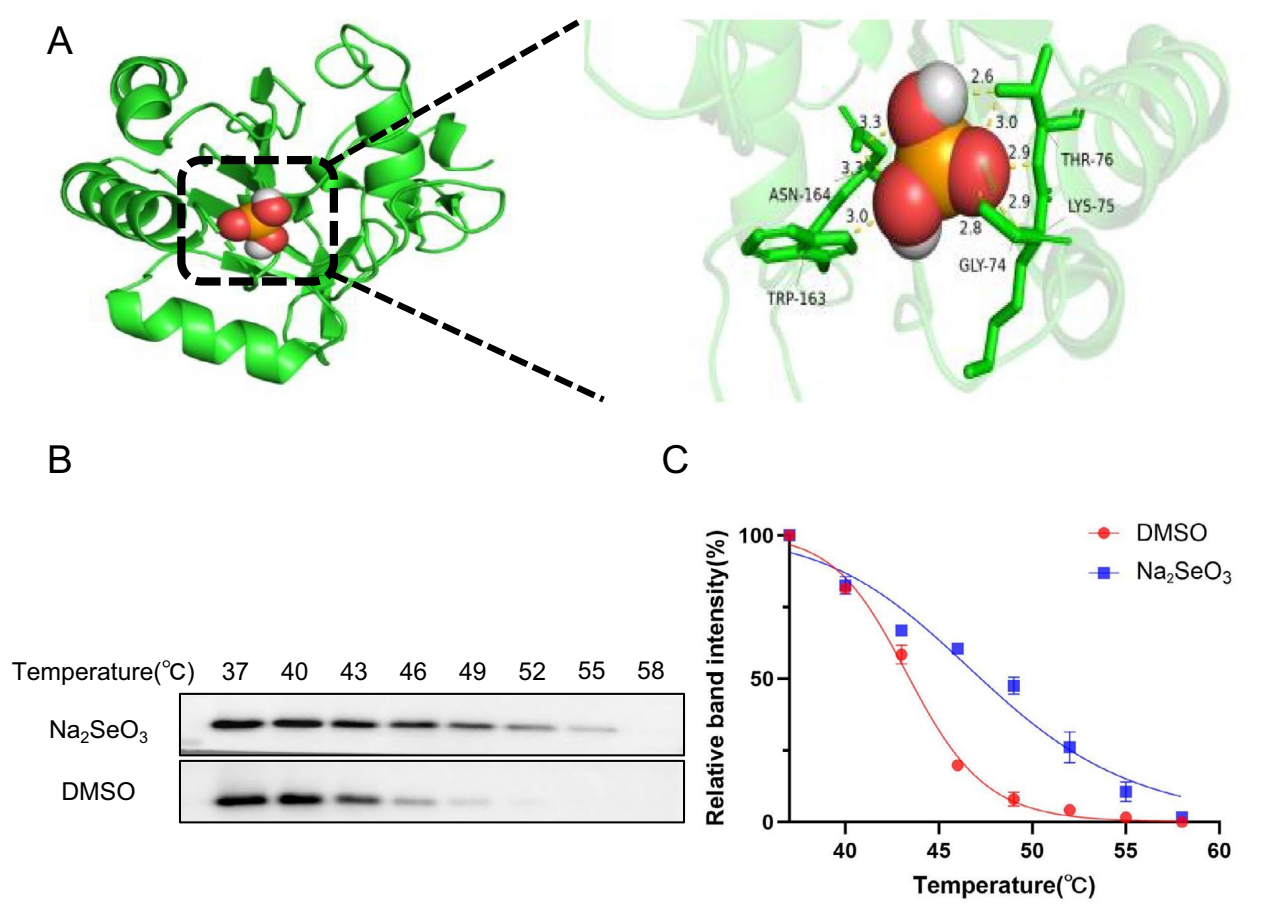
Na_2_SeO_3_ directly targets and stabilises GPX4. (A) Predicted binding interaction between GPX4 and Na_2_SeO_3_. (B) Representative western blot images from the Cellular Thermal Shift Assay (CETSA). (C) Quantification of the CETSA melt curves.

**FIGURE 6:**
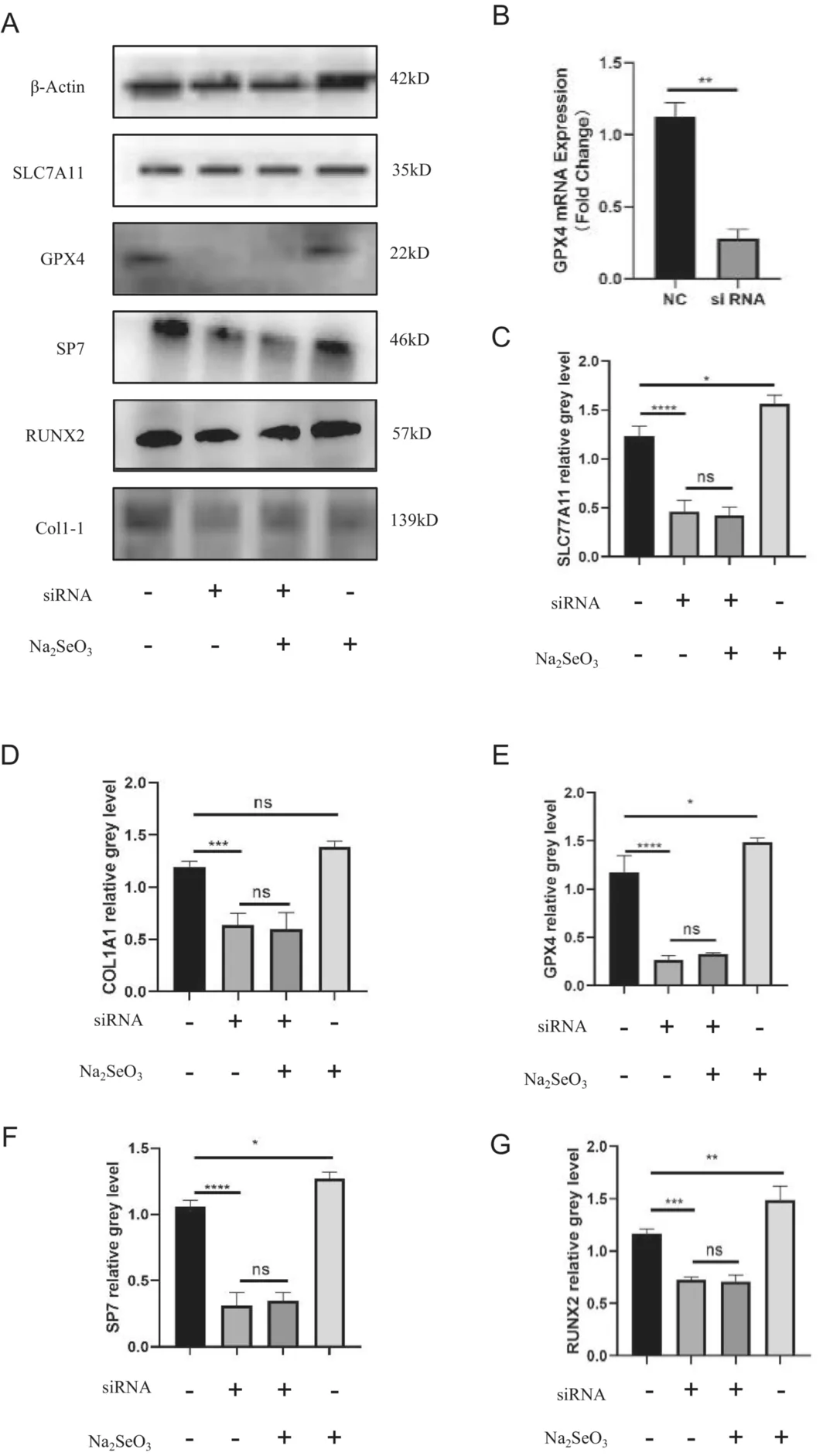
Targeting GPX4 to inhibit ferroptosis. (A) Protein levels of SLC7A11, GPX4, Sp7, RUNX2, and COL1A1. (B) Intracellular GPX4 mRNA expression following GPX4 silencing. (C–G) Quantitative analysis of ferroptosis‐ and osteogenesis‐related proteins shown in (B). Data are expressed as the mean ± SD, *n* = 3; *p < 0.05, **p < 0.01 versus control; ns indicates not significant.

**FIGURE 7:**
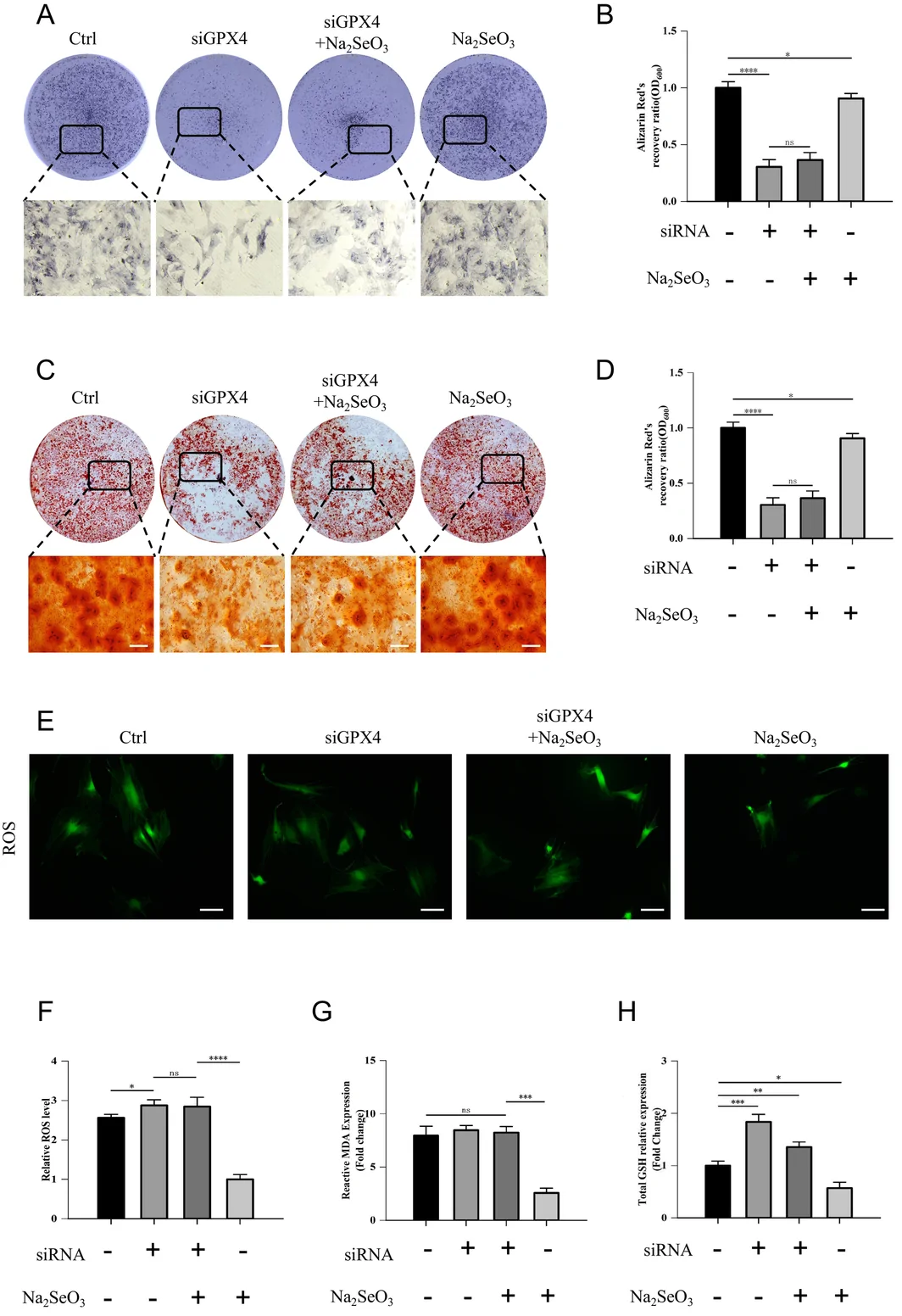
Silencing GPX4 reverses the inhibitory effect of Na_2_SeO_3_ on ferroptosis. (A–C) Representative images of ALP and ARS staining following GPX4 silencing. (B–D) Corresponding quantitative analyses for ALP activity and mineralization. (E–F) Representative images of ROS staining after GPX4 silencing and corresponding quantitative analysis. (G) MDA expression levels after GPX4 silencing. (H) GSH expression levels after GPX4 silencing. All scale bars represent 100 μm. Quantitative data are presented as the mean ± SD, *n* = 3; *p < 0.05, **p < 0.01, ***p < 0.001, ****p < 0.0001 versus control; ns indicates not significant.

Moreover, both ROS and MDA quantification demonstrated that Na_2_SeO_3_ treatment markedly reduced intracellular oxidative stress in BMSCs, whereas the siGPX4 + Na_2_SeO_3_ group did not exhibit a comparable reduction (Figure 7E–G). The GSH assay findings were consistent with the above results, further supporting this conclusion (Figure 7H). Collectively, these results demonstrate that silencing GPX4 completely blocks the ferroptosis‐inhibitory and osteoprotective effects of Na_2_SeO_3_. Thus, GPX4 may represent a key molecular target mediating Na_2_SeO_3_s impacts on both ferroptosis and osteogenic differentiation.

### 4.4 Sodium Selenite Mitigates OVX‐Induced Bone Loss in Vivo

We established an OVX mouse model to assess the role of Na_2_SeO_3_ on osteoporosis. Beginning 1 week after surgery, the mice received daily intraperitoneal injections of Na_2_SeO_3_ at doses of 0.05 mg/kg or 0.1 mg/kg for 14 consecutive days. Micro‐CT analysis and three‐dimensional reconstruction revealed marked trabecular bone loss in the OVX group, whereas Na_2_SeO_3_ treatment significantly increased the trabecular number, with the higher dose producing greater benefits (Figure 8A). Compared with the OVX group, the Na_2_SeO_3_‐treated group showed marked improvements in bone morphometric parameters, indicating a dose‐dependent enhancement of bone formation. Moreover, higher concentrations of Na_2_SeO_3_ were associated with a progressive reduction in bone porosity (Figure 8B–E). Histological evaluation by H&E staining further confirmed that Na_2_SeO_3_ treatment increased trabecular bone quantity and improved its structural organization, with more pronounced effects observed at the higher dose (Figure 8F–I). In addition, Na_2_SeO_3_ promoted new bone formation and enhanced mineral deposition. Quantitative double‐labelling fluorescence with calcein demonstrated increased mean thickness of mineral deposition, further validating these findings (Figure 8G,H).

**FIGURE 8:**
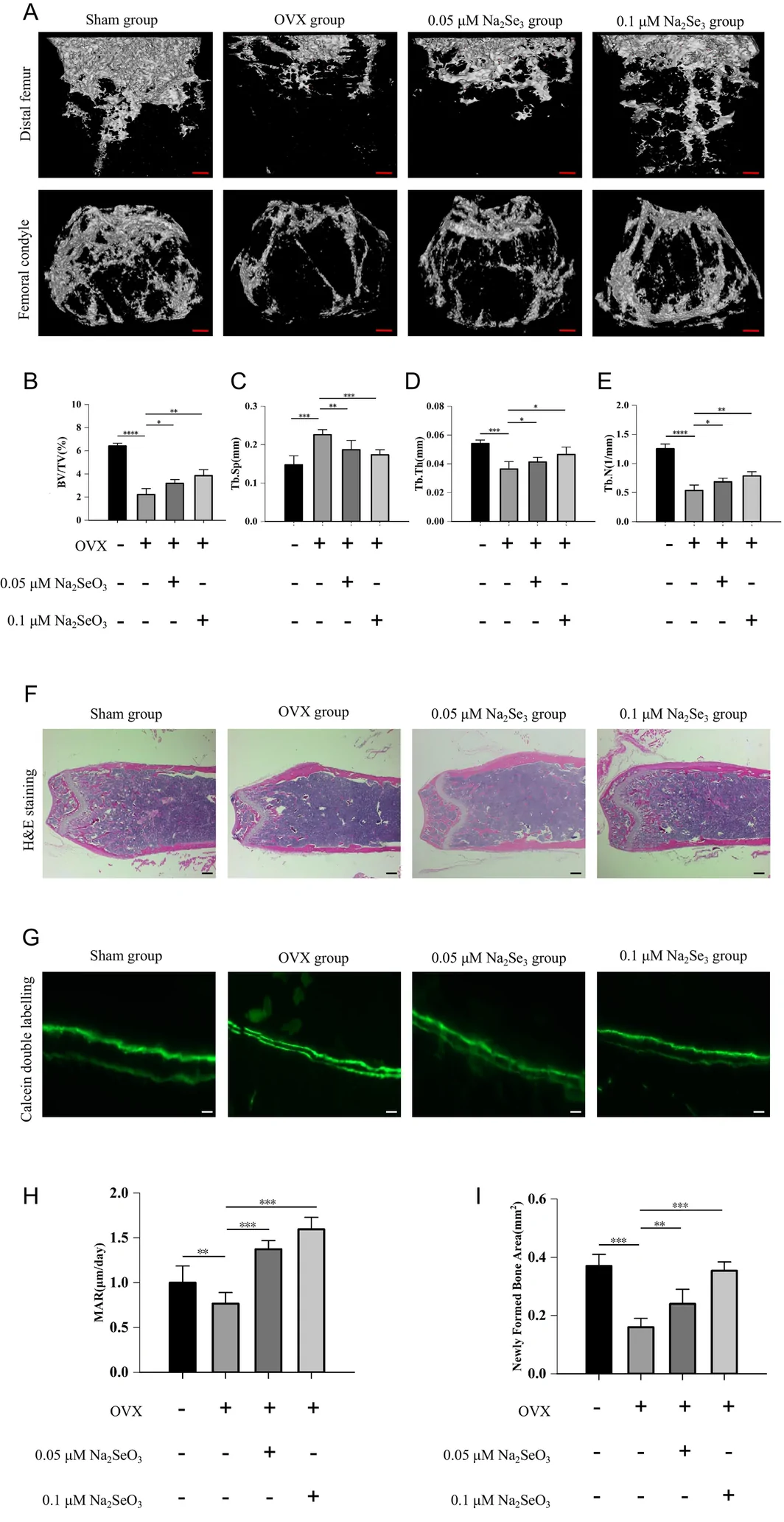
Osteogenic effect of Na_2_SeO_3_ in vivo. (A) Representative three‐dimensional micro‐CT reconstruction images (scale bar = 100 μm). (B) Quantitative analysis of bone volume fraction (BV/TV, %). (C) Trabecular thickness (Tb.Th). (D) Trabecular separation (Tb.Sp), (E) Trabecular number (Tb.N). (F) Representative H&E staining images (scale bar = 50 μm). (D) Calcein double labeling (scale bar = 25 μm). (G) H&E staining of mouse heart, lung, and kidney tissues (scale bar = 50 μm). (H) Calcein labeling for measuring the mineral apposition rate (MAR). (I) Area of newly formed bone shown by HE Staining. Data are expressed as the mean ± SD, *n* = 5; *p <  0.05, **p < 0.01 versus control; ns indicates not significant.

Furthermore, histopathological examination was performed on the major organs of the experimental mice. H&E staining of major organs following Na_2_SeO_3_ treatment revealed no apparent morphological abnormalities (Figure 9A). To further evaluate the systemic biosafety of Na_2_SeO_3_ in vivo, we comprehensively examined serum biochemical indicators of hepatic and renal function. Compellingly, the serum levels of ALT, AST, BUN, and Cr in the Na_2_SeO_3_‐treated groups showed no pathological elevations and remained within normal physiological ranges compared with those in the sham and OVX groups (Figure 9B–E). Collectively, these results suggest that Na_2_SeO_3_ effectively mitigates OVX‐induced bone loss in vivo.

**FIGURE 9:**
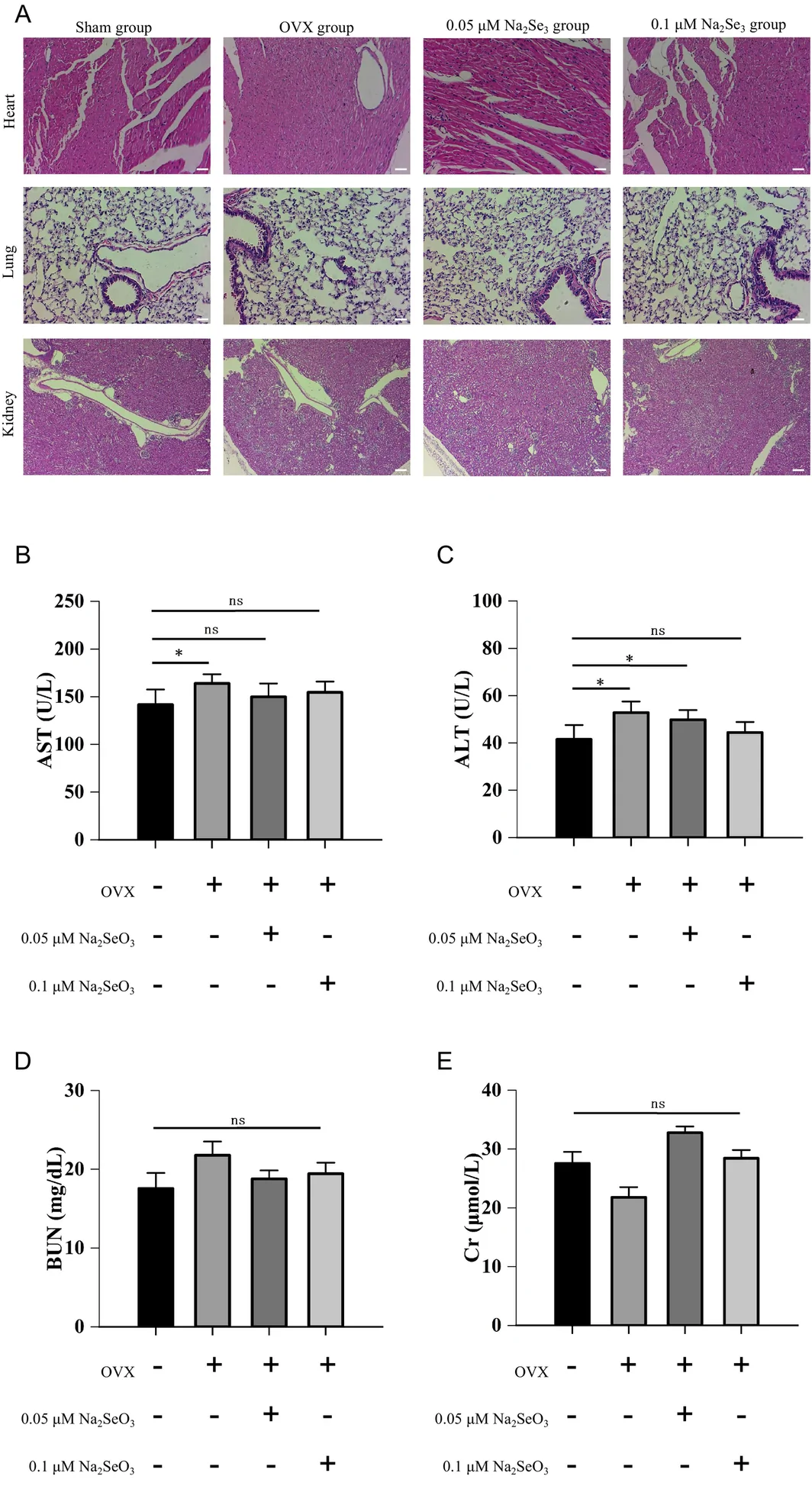
Toxicity of experimental doses of Na_2_SeO_3_ in vivo. H&E staining of mouse heart, lung, and kidney tissues (scale bar = 50 μm). (B–E) Serum biochemical analysis of mice following sodium selenite treatment. The levels of hepatic function markers, including (B) alanine aminotransferase (ALT) and (C) aspartate aminotransferase (AST), as well as renal function markers, including (D) blood urea nitrogen (BUN) and (E) creatinine (Cr), were measured to assess systemic toxicity. Data are expressed as the mean ± SD, *n* = 5; *p < 0.05, **p < 0.01 vs. control; ns indicates not significant.

## 5 Disscusion

Osteoporosis, a systemic pathology of bone metabolism, is defined by a progressive loss of bone mass and the degradation of its trabecular microarchitecture [26]. This structural compromise culminates in diminished skeletal integrity and a consequent elevation in fracture susceptibility [27]. Although the aetiology of osteoporosis is recognised as multifactorial, the condition's central pathogenic driver is a chronic dysregulation of bone remodelling, in which the rate of osteoclastic resorption pathologically exceeds that of osteoblastic bone formation. Current clinical treatments primarily rely on anti‐resorptive agents that inhibit osteoclast activity to slow bone loss [28]; however, their efficacy in promoting new bone formation remains limited [3]. Thus, it is critical to develop therapeutic strategies that actively enhance osteogenesis to optimise osteoporosis management.

Sodium selenite is an inorganic selenium compound and one of the most commonly used selenium supplements in clinical practice [29]. Sodium selenite is an important source of selenium, an essential trace element for humans, and plays a critical role in maintaining antioxidant defence, immune regulation, and thyroid hormone metabolism [30, 31]. However, the therapeutic efficacy of selenium supplementation or selenium‐enriched biomaterials in the direct treatment of osteoporosis remains insufficiently supported by high‐quality evidence‐based studies. Previous research by Lourdes et al. confirmed the key role of selenium in antioxidant protection, immune surveillance enhancement, and cell proliferation regulation [32]. In our study, the impaired osteogenic capacity and elevated ROS fluorescence intensity in H_2_O_2_‐treated BMSCs were effectively mitigated by Na_2_SeO_3_ at 0.05–0.1 μM. OVX model studies combining H&E staining and Micro‐CT analysis further confirmed Na_2_SeO_3_s ability to enhance osteogenic capacity by reducing bone tissue oxidative stress, thereby effectively treating bone loss; however, its clinical application remains limited by inherent toxicity concerns, despite well‐documented pro‐osteogenic effects. Notably, although clinical applications of selenium are sometimes limited by inherent toxicity concerns, our newly incorporated serum biochemical evaluations (ALT, AST, BUN, and Cr) provide compelling evidence that the therapeutic dosage of Na_2_SeO_3_ exhibits excellent systemic biosafety without inducing hepatotoxicity or nephrotoxicity.

Ferroptosis is a distinct modality of programmed cell death precipitated by iron‐dependent, catastrophic lipid peroxidation and is biochemically hallmarked by excessive lipid‐derived ROS accumulation, GSH depletion, and Fe2+ overload [33]. The core mechanism involves dysfunction of the GPX4–SLC7A11 axis, leading to reduced intracellular GSH synthesis and subsequent failure to eliminate lipid ROS, ultimately causing cell membrane disintegration [34]. Yuan X et al. proposed that selenium regulates bone metabolism by modulating ferroptosis in bone tissue cells [15]. Complementing these findings, Yin Y et al. showed that selenium effectively inhibits ferroptosis by elevating GSH levels and SOD enzyme activity, while reducing MDA and iron content, and upregulating GPX4 and SLC7A11 expression [35]. The present study revealed that Na_2_SeO_3_ likely enhances the osteogenic capacity of BMSCs by negatively regulating ferroptosis. To conclusively validate this pathway, we used Ferrostatin‐1 as a positive control. Subsequent phenotypic evaluations confirmed that, as closely mirrored by the pharmacological efficacy of Fer‐1, Na_2_SeO_3_ remarkably suppressed MDA formation, restored GSH levels, and eliminated intracellular labile iron overload. These converging results robustly confirm that Na_2_SeO_3_ rescues the osteogenic potential of BMSCs primarily by acting as a potent ferroptosis inhibitor. GPX4 is a selenoprotein whose catalytic site incorporates selenium in the form of selenocysteine (Sec) [36].

As a central molecular barrier against ferroptosis, GPX4 plays a dual role as both a structural protein and an antioxidant enzyme [37]. Cao et al. reported that GPX4 not only acts as a pivotal regulator of ferroptosis but also participates in lipid and amino acid metabolism, thereby influencing cellular senescence, tumorigenesis, and programmed cell death [38]. Increasing evidence suggests that targeting GPX4 to modulate ferroptosis represents a highly promising therapeutic strategy. In the present study, silencing GPX4 via siRNA markedly impaired the osteogenic capacity of BMSCs, accompanied by decreased expression of ferroptosis‐related proteins GPX4 and SLC7A11. These findings demonstrate that Na_2_SeO_3_ promotes the osteogenic differentiation of BMSCs by upregulating GPX4 expression, thereby suppressing ferroptosis.

However, the precise mechanisms underlying Na_2_SeO_3_‐induced GPX4 upregulation remain unclear. Beyond the physical binding suggested by molecular docking, Na_2_SeO_3_ likely regulates GPX4 multidimensionally. Na_2_SeO_3_ may post‐translationally stabilize the GPX4 protein against proteasomal degradation under oxidative stress, while also translationally enhancing GPX4 synthesis by supplying selenium for selenocysteine incorporation. Furthermore, Na_2_SeO_3_ could transcriptionally activate the Nrf2/Keap1 axis to co‐upregulate GPX4 and SLC7A11 expression, forming a synergistic regulatory network that underpins its robust anti‐ferroptotic efficacy.

Despite these promising findings, the current study has certain limitations. Relying solely on an H_2_O_2_‐induced in vitro model may not fully capture the multifactorial aetiology of osteoporosis, necessitating future validations in alternative models such as glucocorticoid‐induced or age‐related osteoporosis. Additionally, the short observation period in our OVX animal model underscores the need for longitudinal studies to assess the long‐term therapeutic durability and systemic biosafety of Na_2_SeO_3_. Furthermore, since we focused exclusively on BMSC osteogenesis, evaluating Na_2_SeO_3_s direct impact on osteoclast‐mediated bone resorption remains a critical next step to fully elucidate its dual‐target clinical potential.

## 6 Conclusion

This study demonstrates that Na_2_SeO_3_ treatment significantly attenuates intracellular ROS accumulation in BMSCs under oxidative stress conditions while enhancing their osteogenic differentiation capacity. Further mechanistic investigations reveal that GPX4, functioning as a selenium‐dependent key regulator, governs cell survival and osteogenic function by suppressing the ferroptosis pathway. To assess the translational potential of these results, we investigated the effects in an in vivo setting. In the OVX mouse model, sodium selenite similarly exhibited efficacy in mitigating bone loss. The convergence of these results demonstrates that sodium selenite's protective action is mechanistically rooted in the suppression of osteoblast ferroptosis, thus validating its potential as a targeted therapeutic intervention for osteoporosis.

## Author Contributions

**Wei Luo:** conceptualization, investigation, formal analysis, visualization, writing – original draft. **Haibo Xu:** investigation, software, writing – review and editing. **Xin Liu:** investigation, software, writing – review and editing. **Yongsheng Zhang:** data curation, investigation. **Hao Zhou:** data curation, investigation, writing – review and editing. **Xiangqiang Pan:** conceptualization, supervision, project administration, writing – review and editing. **Dechun Geng:** conceptualization, supervision, project administration. **Hanliang He:** conceptualization, supervision, project administration, writing – review and editing.

## Funding

This work was supported by 2025 Suzhou Medical and Industrial Cooperation Innovation Project, SZM2025018.

## Ethics Statement

All animal procedures were conducted under an approved protocol (No. 2023231033) at the Fourth Affiliated Hospital of Soochow University.

## Consent

The authors have nothing to report.

## Conflicts of Interest

The authors declare no conflicts of interest.

## Supporting information


**Table S1:** All primer sequences used in qRT‐PCR.
**Table S2:** Sequences for the siRNA.

## Data Availability

The data that support the findings of this study are available from the corresponding author upon reasonable request.
