# Supplementary material for: GPX4‐Mediated Inhibition of Ferroptosis by Sodium Selenite Ameliorates Bone Metabolic Disorders in Ovariectomized Mice
Source: J Cell Mol Med. 2026 Oct 19;30(20):e71414. doi: 10.1111/jcmm.71414 (PMC13678664; doi:10.1111/jcmm.71414)
Supplement: Supplementary file 1 [file JCMM-30-e71414-s001.docx]

S1 All primer sequences used in qRT-PCR

| **Gene** | **Primer Sequence F** | **Primer Sequence R** |
| --- | --- | --- |
| RUNX2 | GGTGAAACTCTTGCCTCGTC | AGTCCCAACTTCCTGTGCT |
| OCN | CAGGGAGGCAGTGACTCTTC | AGTGTGGAAAGTGTGGAGTT |
| SP7 | TGGTACAAGGCAGGCATCCA | GGAGCAAAGTCAGATGGGTAAGT |
| GPX4 | GCGGGCTACAACGTCAAATTC | GCAGGTCCTTCTCTATCACCAG |
| SLC7A11 | CTTTCAAGGTGCCACTGTTCATC | ACGAAGCCAATCCCTGTACTAAA |
| PTGS2 | CTGGCGCTCAGCCATACAG | CGCACTTATACTGGTCAAATCCC |
| β-actin | CAGCCTTCCTTCTTGGGTATG | AGCTCAGTAACAGTCCGCCT |

S2 Sequences for the siRNA

| **Gene** | **Sequence** | |
| --- | --- | --- |
|  | **sense（5'-3'）** | **antisense（5'-3'）** |
| Gpx4-Mus-79 | CCGUCUGAGCCGCUUACUUTT | AAGUAAGCGGCUCAGACGGTT |
